# Efficacy and safety of intralesional triamcinolone acetonide alone and its combination with 5‐ fluorouracil in keloids and hypertrophic scars: Randomized, parallel group, and double blinded trial

**DOI:** 10.1002/ski2.450

**Published:** 2024-08-24

**Authors:** Ripala Acharya, Sudha Agrawal, Dhan Keshar Khadka, Aashish Raj Pant

**Affiliations:** ^1^ Department of Dermatology and Venereology B.P Koirala Institute of Health Sciences Dharan Nepal; ^2^ Department of Ophthalmology Maharajgunj Medical Campus Tribhuwan University Kathmandu Nepal

## Abstract

**Background:**

Treatment of keloids and hypertrophic scars is challenging. The current first‐line treatment is a steroid which has high resistance and recurrence rate along with unacceptable adverse effects. Different studies involving the combination of TAC and 5‐FU that have been done so far showed better treatment outcomes in terms of efficacy and safety.

**Objective:**

The objective of this study was to compare the efficacy and safety of intralesional triamcinolone acetonide alone and its combination with 5‐fluorouracil in patients with keloids and hypertrophic scars at 12 weeks follow‐up.

**Methods:**

In this randomized parallel group double‐blinded clinical trial, we enroled 66 cases of keloids and hypertrophic scars randomly allocated into two treatment groups. Patients received an intralesional injection of triamcinolone acetonide (20 mg/mL) in Group A and an intralesional injection of a combination of triamcinolone acetonide (20 mg/mL) and 5‐fluorouracil (25 mg/mL) in Group B for every 2 weeks until 10 weeks and the final evaluation was done at 12 weeks follow‐up.

**Results:**

There was a reduction in all the parameters at every follow‐up visit in both groups. The ≥50% reduction in height, reduction in the VSS and POSAS scores, and good to excellent subjective improvement reported by both the patients and the observer were significantly greater in the combination group compared to TAC alone group. The response rate was faster and complications were lesser in the combination group as compared to TAC alone group.

**Limitation:**

Single‐centred, no long‐term follow‐up, and recurrence could not be assessed.

**Conclusion:**

TAC alone and its combination with the 5‐FU both were effective in keloids and hypertrophic scars. Yet, the TAC and 5‐FU combination treatment was more efficacious with a faster response rate and safer than the TAC alone treatment.

## Graphical abstract

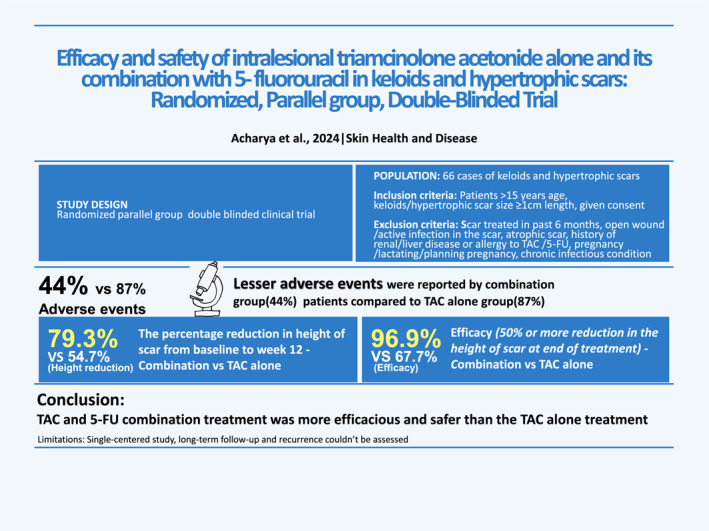

**What's already known about this topic?**

- Combination of triamcinolone acetonide and 5‐fluorouracil has shown promising results in the literature. To date, intralesional triamcinolone acetonide and 5‐fluorouracil in a 1:1 combination has been rarely studied and no definite treatment protocol is available.

**What does this study add?**

- This study supports that both the intralesional triamcinolone acetonide alone and combination with 5‐fluorouracil (1:1) are effective for the treatment of pathological scars with the latter being significantly superior in terms of efficacy with faster response rate and reduced side effects.

**Abbreviations**
5‐FU: 5‐fluorouracil
CI: confidence interval
IRC: Institute Research Committee
MD: mean deviation
NHRC: Nepal Health Research Council
POSAS: The Patient and Observer Scar Assessment Score RCT Randomized Controlled Trial
SD: standard deviation
SPSS: statistical package for social sciences
TAC: triamcinolone acetate
VSS: Vancouver scar scale

## 1 INTRODUCTION

Keloids and hypertrophic scars are problematic scars with a burden on the health care system and the functional and psychological well‐being of an individual.^1^ The current first‐line therapy is an intralesional triamcinolone (TAC) injection, but approximately 50% of keloids are steroid‐resistant.^2^ High proportions of keloids either do not respond to TAC or develop a recurrence and approximately half of the TAC‐treated keloids are associated with side effects.^3^ Thus, newer therapeutic options like antineoplastic agent 5‐ fluorouracil (5‐FU) have emerged as viable options. The use of antineoplastic agents is logical because these abnormal tissues are in a hyperproliferative state.^4^ Preliminary studies had shown better efficacy of TAC and 5‐FU combination over the TAC alone but lack a definite protocol regarding dosing and frequency.^5^, ^6^, ^7^, ^8^, ^9^, ^10^, ^11^, ^12^, ^13^ Recent meta‐analyses have recommended additional randomized, controlled, large‐sample, and high‐quality trials for the objective analysis of treatment efficacy and associated adverse reaction.^7^, ^14^ Here, we took a low concentration of 5‐FU and a higher concentration of TAC at 2 weeks interval^15^ to compare the efficacy and safety profile of intralesional triamcinolone acetonide alone and its combination with 5‐FU in the treatment of keloids and hypertrophic scars.

## 2 MATERIALS AND METHODS

### 2.1 Study design

We conducted a randomized parallel group double‐blinded clinical trial in the setting of the Department of Dermatology and Venereology at the B.P. Koirala Institute of Health Sciences (BPKIHS) after the ethical approval from the Institutional Review Committee, BPKIHS (Code no: IRC/1890/020) and Nepal Health Research Council (ERB Protocol No:721/2020 MT). The trial was registered in ClinicalTrial.gov (NCT04812626). Written, well‐understood, and signed informed consent form (for patients aged 18 years or old) and assent form (patients age 16–17 years) were obtained.

### 2.2 Study population

Inclusion criteria included: patients >15 years age, keloid or hypertrophic scar of size ≥1 cm length, and consent for participation.

Patients who took treatment for the same scar in the past 6 months had an open wound or active infection in the scar or atrophic scar, had a history of renal disease or altered liver enzymes or white blood cell count or a history of allergy to 5‐fluorouracil or triamcinolone acetonide, immunocompromised status or patients with pregnancy, lactation or planning for pregnancy were excluded. Co‐existing chronic infectious and granulomatous conditions like tuberculosis were also excluded.

### 2.3 Recruitment and randomisation of participants

Patients were recruited from January 2021 to August 2021 from a single outpatient department of Dermatology and Venereology, BPKIHS. Detailed information and examination findings of the patients satisfying the above criteria were recorded in a Proforma. Photographs of the lesion were taken before the treatment, during each treatment sessions, and at the 12th week. Only one lesion, preferably trunk or proximal extremity was treated per patient with multiple lesions.

Each enroled patient was randomly allocated to one of the two treatment groups (Group A and Group B) using a block randomisation list of block sizes 4, 6, and 8 and a seed of 110488575778059 to produce two parallel groups (1:1 ratio) of patients with the help of www.sealedenvelope.com. A sequentially generated number with the treatment group was written in a sealed envelope and prepared by the independent dermatologist before the enrolment of patients.

### 2.4 Blinding

Both patient and trial investigator.

### 2.5 Interventions

Scars in Group A received intralesional TAC alone where 1 mL of TAC (40 mg/mL) was mixed with 1 mL of 0.9% normal saline (final concentration 20 mg/mL) and Group B received an intralesional TAC and 5‐FU combination where 1 mL of TAC (40 mg/mL) was mixed with 1 mL of 5‐FU (50 mg/mL), the final concentration of TAC and 5‐FU being 20 mg/mL and 25 mg/mL, respectively.

The mixture was injected intralesionally using a 40‐unit insulin syringe with a 27‐G needle in the keloid/hypertrophic scar lesion until a slight blanching was seen clinically after the field block with 2% xylocaine. This approximates to 3–7 mm according to the size of the lesion. An injection was given using the grid method as explained by.

E. Agius et al.^16^ and Camacho Martinez et al.^17^ Five units (0.125 mL) of the mixture was given intralesional with the help of 1 mL insulin syringe of 40units, that covered an area of about 0.5 × 0.5 cm^2^ of scar, thus, a scar of 1 cm^2^ required 4 injections to cover the whole lesion. The injection was given into the body of the scar till slight blanching with subsequent injection sites being 5 mm apart. For the larger lesions, the dose was increased upto 2 mL in each group (maximum dose of TAC/5‐FU: 40 mg/50 mg). The injection was given every 2 weeks for 6 doses. The final evaluation of the scar was done in week 12.

### 2.6 Outcome measurements

Primary outcomes compared between the two groups from baseline to week 12 were as follows: 1. Reduction in the height of the scar, 2. mean change in the POSAS score, 3. mean change in the VSS score, and 4. subjective improvement: good to excellent (>50%) at 12 weeks from the baseline (patient and observer). Parameters like height, VSS score, and POSAS score were noted at each visit. The overall improvement was graded by the patient and observer separately on a 5‐point scale and the percentage of improvement was assessed in each follow‐up visit (no/poor: up to 25%, fair: 26%–50%, good: 51%–75%, and excellent: 76%–100% improvement).

The secondary outcome measures were the side effects of drugs like pigmentation, ulceration, atrophy, and telangiectasia.

### 2.7 Sample size

Based on study by **Darougegh A et al. (2009)**,^18^ the number of patients who reported good to excellent improvement in the TAC alone group were 20% and in the combination of TAC and 5‐FU were 55%. Assuming 95% confidence interval, 80% power, and 10% loss to follow‐up, the total sample size was 66 (33 in each treatment group) using the sample size estimation formula for 2 proportions.

### 2.8 Efficacy evaluation

Efficacy was considered as a 50% or more reduction in the initial height or more than 50% subjective improvement as reported by the patient or observer at 12 weeks.

### 2.9 Safety evaluation

Safety of treatment was evaluated through local adverse effects like erythema, pain, atrophy, ulceration, telangiectasia, pigmentation, and systemic effects if any.

### 2.10 Statistical analysis

Data were analysed in SPSS version 25. Statistical analyses were conducted on both per‐protocol (PP) and intention‐to‐treat (ITT) population (defined as all enroled patients to whom the study drug was given; with the last observation carried forward) basis using two‐sided tests. Statistical methods used were the Chi‐square test, Paired *t*‐test, Independent *t*‐test, Wilcoxon signed rank test, Mann–Whitney *U* test and Kaplan–Meier curves. The test of significance was considered when the value was *p* < 0.05.

## 3 RESULTS

A total of 66 patients were enrolled (33 in each group). Seven patients could not complete the study due to the ongoing COVID‐19 pandemic (two from TAC alone and one from the combination could not follow up after the initial visit), hence they were excluded from the final analyses. Hence, 59 patients completed the study (29 from the TAC alone group and 30 from the combination group). Baseline characteristics of patients in both groups were comparable (Supplementary Table S1).

### 3.1 Reduction in height

In the ITT analysis, the mean baseline height in the TAC alone group was 7.00 ± 3.06 mm (median: 6 mm), reduced by 13.7%, 24.8% 34.3%, 39.4%, 47.3%, and 54.2% at weeks 2, 4, 6, 8, 10, and 12 respectively. In the combination group, the mean height at baseline was 5.41 ± 1.68 mm (median: 5 mm), reduced by 34.2%, 49.7%, 60.2%, 66.8%, 73.7%, and 77.7% at weeks 2, 4, 6, 8, 10, and 12 respectively. Similar results were obtained in the PP analysis. (Supplementary Figure S2).

There was a significant decrease in the height of lesions in both the treatment groups at all weeks compared to the baseline (*p* < 0.05) in both the ITT and PP population. The percentage reduction in height from the baseline to week 12 between the treatment groups was greater in the TAC + 5‐FU group than in the TAC alone group in both the ITT (77.7% vs. 54.2%) and PP (79.3% vs. 54.7%) population (*p* < 0.05). Similarly, in week 12, 50% or more reduction in the height was found significantly more in the TAC + 5‐FU group than in TAC alone group in both the ITT (96.9% vs. 67.7%) and PP population (100% vs. 72.4%) (*p* = 0.002) (Table 1).

**TABLE 1:** Comparison of efficacy (>/ = 50% reduction in height) between the treatment groups in each follow‐up visits.

Week	Efficacy	ITT population		PP population	
		TAC alone (*N* = 31) *n* (%)	TAC + 5‐FU (*N* = 32) *n* (%)	TAC alone (*N* = 29) *n* (%)	TAC + 5‐FU (*N* = 30) *n* (%)
Week 2	Yes	1 (3.22)	9 (28.12)	1 (3.45)	8 (26.67)
	No	30 (96.78)	23 (71.88)	28 (96.55)	22 (73.33)
	*p*‐value^a^	0.007		0.015	
Week 4	Yes	4 (12.9)	19 (59.38)	4 (13.79)	18 (60)
	No	27 (87.1)	13 (40.62)	25 (86.21)	12 (40)
	*p*‐value^a^	<0.001		<0.001	
Week 6	Yes	9 (29.03)	27 (84.38)	9 (31.03)	26 (86.67)
	No	22 (70.97)	5 (15.62)	20 (68.97)	4 (13.33)
	*p*‐value^a^	<0.001		<0.001	
Week 8	Yes	14 (45.16)	30 (93.75)	14 (48.275)	29 (96.67)
	No	17 (54.84)	2 (6.25)	15 (51.725)	1 (3.33)
	*p*‐value^a^	<0.001		<0.001	
Week 10	Yes	19 (61.29)	31 (96.88)	19 (65.51)	30 (100)
	No	12 (38.71)	1 (3.12)	10 (34.49)	0 (0)
	*p*‐value^a^	<0.001		<0.001	
Week 12	Yes	21 (67.74)	31 (96.88)	21 (72.41)	30 (100)
	No	10 (32.26)	1 (3.12)	8 (27.59)	0 (0)
	*p*‐value^a^	0.002		0.002	

^a^ Pearson chi‐square test.

**FIGURE 1:**
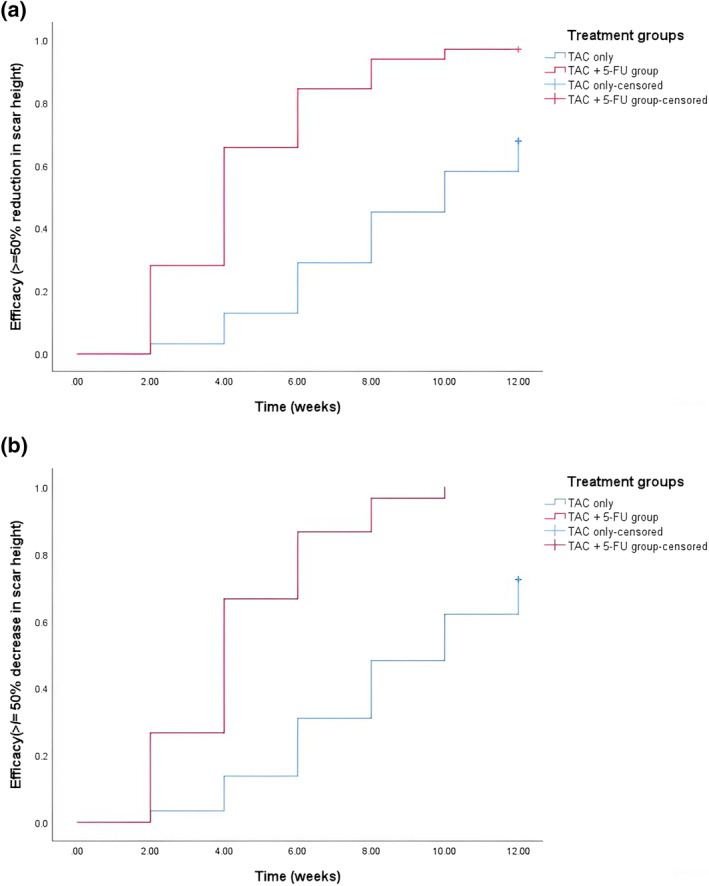
Kaplan–Meier survival analysis for 50% or more reduction in height inbetween the 2 groups: (i) ITT and (ii) PP populations.

Kaplan–Meier analysis suggested that the 12‐week cumulative probability of achieving ≥50% reduction in initial scar height was significantly (*p* < 0.001) higher in the TAC + 5‐FU group compared to the TAC alone group in both the ITT (96.9% vs. 67.7%) and PP (100% vs. 72.4%) population. The median time for effect in the TAC alone group was 10 weeks, whereas it was only 4 weeks in the TAC + 5‐FU group (Figure 1).

### 3.2 Reduction in VSS scores

VSS scores were reduced significantly at week 12 compared to baseline in both treatment groups (*p* < 0.0001) (both ITT and PP). There was no significant difference in the VSS score at the baseline between the groups (*p* = 0.784) but at 12 weeks follow‐up, it was significantly lower in the combination group compared to the TAC alone group (*p* < 0.05) in both the ITT and PP population (Table 2).

**TABLE 2:** Comparison of VSS score (0–13), observer POSAS (6–60), and patient POSAS (6–60) in between the treatment groups.

		ITT population						PP population					
		VSS		Observer POSAS		Patient POSAS		VSS		Observer POSAS		Patient POSAS	
Week (wk)		TAC alone, *N* = 31	TAC + 5‐FU, *N* = 32	TAC alone, *N* = 31	TAC + 5‐FU, *N* = 32	TAC alone, *N* = 31	TAC + 5‐FU, *N* = 32	TAC alone, *N* = 29	TAC + 5‐FU, *N* = 3 0	TAC alone, *N* = 29	TAC + 5‐FU, *N* = 30	TAC alone, *N* = 29	TAC + 5‐FU, *N* = 30
Wk 0	Mean ± SD (median)	9.06 ± 1.65 (9)	9.22 ± 0.90 (9)	33.55 ± 8.74 (35)	34.59 ± 5.40 (33)	33.74 ± 8.43 (34)	37.69 ± 8.68 (37.5)	9.03 ± 1.70 (9)	9.23 ± 0.93 (9)	32.93 ± 8.41 (35)	34.67 ± 5.54 (33)	33.41 ± 8.58 (32)	38.03 ± 8.79 (38.5)
	*p*‐value^a^	0.655		0.923		0.144		0.784		0.682		0.88	
Wk 2	Mean ± SD (median)	8.87 ± 1.23 (9)	8.50 ± 0.71 (9)	31.483 ± 8.25 (32)	28.03 ± 5.78 (27)	28.26 ± 8.31 (28)	23.78 ± 7.48 (23)	8.83 ± 1.25 (9)	8.50 ± 0.73 (9)	30.83 ± 7.76 (31)	28.27 ± 5.83 (27)	27.66 ± 8.15 (27)	24.13 ± 7.57 (24)
	*p*‐value^a^	0.016		0.029		0.030		0.033		0.078		0.092	
Wk 4	Mean ± SD (median)	8.55 ± 1.45 (9)	7.94 ± 1.24 (8)	27.45 ± 8.29 (29)	23.00 ± 6.42 (25)	24.48 ± 7.59 (25)	17.97 ± 4.73 (18)	8.48 ± 1.47 (9)	7.9 ± 1.27 (8)	26.93 ± 8.29 (28)	22.90 ± 6.56 (23)	24.07 ± 7.67 (25)	17.93 ± 4.84 (18)
	*p*‐value^a^	0.044		0.005		0.000		0.071		0.011		0.001	
Wk 6	Mean ± SD (median)	8.26 ± 1.5 7 (8)	7.34 ± 1.28 (7.5)	26.61 ± 7.37 (26)	21.84 ± 4.60 (22)	21.74 ± 7.80 (20)	15.53 ± 3.65 (14.5)	8.17 ± 1.58 (8)	7.27 ± 1.28 (7)	26.03 ± 7.23 (26)	21.67 ± 4.62 (22)	21.14 ± 7.69 (20)	15.33 ± 3.63 (14)
	*p*‐value^a^	0.009		0.002		0.000		0.013		0.003		0.001	
Wk 8	Mean ± SD (median)	8.23 ± 1.78 (9)	6.69 ± 1.4 (7)	25.00 ± 6.77 (26)	19.88 ± 4.98 (20)	20.65 ± 7.06 (20)	14.06 ± 3.80 (14)	8.14 ± 1.80 (8)	6.57 ± 1.3 6 (6.5)	24.31 ± 6.3 9 (25)	19.57 ± 4.92 (19.5)	19.97 ± 6.76 (20)	13.77 ± 3.69 (14)
	*p*‐value^a^	<0.001		0.001		0.000		<0.001		0.002		<0.001	
Wk 10	Mean ± SD (median)	7.90 ± 1.73 (8)	6.09 ± 1.85 (6)	22.94 ± 6.82 (23)	17.50 ± 5.06 (17.5)	16.39 ± 6.21 (17)	12.31 ± 3.69 (12)	7.79 ± 1.74 (8)	5.93 ± 1.7 9 (6)	22.10 ± 6.18 (23)	17.03 ± 4.79 (16.5)	17.55 ± 5.47 (17)	11.90 ± 3.37 (11.5)
	*p*‐value^a^	<0.001		0.001		0.000		<0.001		0.001		<0.001	
Wk 12	Mean ± SD (median)	7.58 ± 1.74 (8)	5.81 ± 1.87 (6)	21.94 ± 6.63 (23)	16.63 ± 5.19 (16.5)	17.84 ± 5.79 (17)	11.28 ± 3.62 (11)	7.45 ± 1.72 (8)	5.63 ± 1.79 (6)	21.03 ± 5.79 (22)	16.10 ± 4.84 (16)	16.97 ± 5.13 (16)	10.80 ± 3.12 (10.5)
	*p*‐value^a^	<0.001		0.001		0.000		<0.001		0.001		<0.001	

^a^ Mann Whitney Test.

### 3.3 Reduction in POSAS scores (observer and patient)

Findings in both the ITT and PP populations were similar. Both observer and patient POSAS scores reduced significantly at week 12 compared to baseline in both the treatment groups (*p* < 0.0001). There was no statistical difference in both observer and patient POSAS scores at the baseline between the groups (*p* > 0.05) but in the 12 weeks follow‐up, both observer and patient POSAS scores were significantly lower in the combination group compared to the TAC alone group (*p* < 0.05 Table 2).

### 3.4 Subjective improvement (patient and observer)

Both the patient and observer reported that good to excellent subjective improvement were significantly greater in the combination group in the 12‐week follow‐up visits than in the TAC alone group (*p* < 0.05) in both the ITT and PP population (Table 3).

**TABLE 3:** Patient and observer reported good–excellent subjective improvement (>50% improvement).

Week (wk)	>50% improv ement	ITT population				PP population			
		Patient reported improvement		Observer reported improvement		Patient reported improvement		Observer reported improvement	
		TAC alone, *N* = 31 *n* (%)	TAC + 5‐FU, *N* = 32 *n* (%)	TAC alone, *N* = 31 *n* (%)	TAC + 5‐FU, *N* = 32 *n* (%)	TAC alone, *N* = 29 *n* (%)	TAC + 5‐FU, *N* = 30 *n* (%)	TAC alone, *N* = 29 *n* (%)	TAC + 5‐FU, *N* = 30 *n* (%)
Wk 2	Yes	2 (6.45)	12 (37.5)	0 (0)	3 (9.4)	2 (6.9)	11 (36.7)	0 (0)	3 (10)
	No	29 (93.55)	20 (62.5)	31 (100)	29 (90.6)	27 (93.1)	19 (63.3)	29 (100)	27 (90)
	*p*‐value^a^	0.003		0.081		0.006		0.080	
Wk 4	Yes	6 (19.35)	27 (84.38)	1 (3.23)	18 (56.25)	6 (20.7)	26 (86.7)	1 (3.4)	18 (60)
	No	25 (80.65)	5 (15.62)	30 (96.77)	14 (43.75)	23 (79.3)	4 (13.3)	28 (96.6)	12 (40)
	*p*‐value^a^	<0.001		<0.001		<0.001		<0.001	
Wk 6	Yes	11 (35.48)	30 (93.75)	3 (9.68)	26 (81.25)	11 (37.9)	29 (96.7)	3 (10.3)	26 (86.7)
	No	20 (64.52)	2 (6.25)	28 (90.32)	6 (18.75)	18 (62.1)	1 (3.3)	26 (89.7)	4 (13.3)
	*p*‐value^a^	<0.001		<0.001		<0.001		<0.001	
Wk 8	Yes	14 (45.16)	30 (93.75)	5 (16.13)	28 (87.5)	14 (48.3)	29 (96.7)	5 (17.2)	28 (93.3)
	No	17 (54.84)	2 (6.25)	26 (83.87)	4 (12.5)	15 (51.7)	1 (3.3)	24 (82.8)	2 (6.7)
	*p*‐value^a^	<0.001		<0.001		<0.001		<0.001	
Wk 10	Yes	17 (54.84)	30 (93.75)	12 (38.7)	28 (87.5)	17 (58.6)	29 (96.7)	12 (41.4)	28 (93.3)
	No	14 (45.16)	2 (6.25)	19 (61.3)	4 (12.5)	12 (41.4)	1 (3.3)	17 (58.6)	2 (6.7)
	*p*‐value^a^	<0.001		<0.001		<0.001		<0.001	
Wk 12	Yes	18 (58.06)	30 (93.75)	15 (48.39)	29 (90.63)	18 (62.1)	29 (96.7)	15 (51.7)	29 (96.7)
	No	13 (41.94)	2 (6.25)	16 (51.61)	3 (9.37)	11 (37.9)	1 (3.3)	14 (48.3)	1 (3.3)
	*p*‐value^a^	0.001		<0.001		0.001		<0.001	

^a^ Pearson chi‐square test.

### 3.5 Adverse effects

Overall, 41 out of 63 patients developed adverse effects. In the TAC alone group, 27 (87%) developed adverse effects whereas in the combination group only 14 (44%) developed adverse effects (*p* = 0.000). (Supplementary Table S2) Hyperpigmentation was seen in 45.2% cases in the TAC alone group and 40.6% in the combination group. (*p* = 0.716). Approximately 52% in the TAC alone group and 3.1% in the combination group developed telangiectasia which was statistically significant (*p* < 0.001). A single patient in the combination group developed pain post‐treatment. No systemic adverse effects were seen and the local side effects were tolerable.

## 4 DISCUSSION

The most common conventional treatment by corticosteroids is used in variable doses of 10–40 mg/mL at variable time intervals ranging from 2 to 4 weeks. The reported success rate is 50%–100% with adverse effects like skin atrophy, pigmentation, and telangiectasia.^19^

The pathological scars are in a hypermetabolic state with over‐activation of fibroblasts leading to abnormal collagen deposition.^4^ 5‐FU with antimetabolite activity inhibits fibroblasts, hence its use in keloids and hypertrophic scars is logical.^20^ 5‐FU is a safe intralesional steroid with no systemic side effects but with redness and ulceration in some cases. TAC can be added to minimise its side effects. Several studies have shown better efficacy and safety profile of combination therapy of TAC and 5‐FU as compared to TAC or 5‐FU alone.^12^, ^18^ However, there is no consensus regarding the dosage and the frequency.

Regarding the dosage, Coppola MM et al. advocated a dose of 10–40 mg/mL of TAC to have any therapeutic effect in keloids or hypertrophic scar.^6^ We took the middle way with 20 mg/mL TAC concentration where TAC not only decreases the 5‐FU side effects but also increases the therapeutic efficacy of the combination treatment. Our study used a lower concentration of 5‐FU, that is, 25 mg/mL as it would not compromise the efficacy but rather curtail the side effects of 5‐FU. Similarly, we devised our dosing protocol based on the study by Aluko‐Olokun B et al. which found that the response rate of keloids and hypertrophic scar to TAC is high in the first 2 weeks after which the response decreases.^15^ Hence, we took a 2‐week dosing regimen. Treatment duration was taken as 12 weeks as per previous studies and thus, 6 follow‐ups were done.^11^, ^18^, ^19^

In the study conducted by **F.A. Khalid et al. 2018**, 49% in TAC alone group and 77.2% in TAC + 5‐FU combination group showed ≥50% reduction in initial height (*p* = 0.02).^19^ Similarly, **Khan MA et al. (2014)** found ≥50% reduction in the height in 68% of the TAC alone group and 84% in the combination group.^11^ The higher efficacy based on the height reduction in our study can be explained by the higher concentration of TAC, that is, 20 mg/mL.

Our study showed that both the patient and observer reported good to excellent subjective improvement significantly more in the combination group than in the TAC alone group in a 12‐week follow‐up. In the study by **Darougheh A. et al. (2007)**, patients reported good to excellent (>50%) improvement in 20% in TAC alone and 55% in the TAC + 5‐FU group at the final assessment, which was significant (*p* = 0.02).^18^ However, the observer reported good to excellent (>50%) improvement was 15% in the TAC alone group and 40% in the TAC + 5‐FU group which was not significantly significant.^18^

Our study showed significant reduction in the VSS score in the combination group compared to the TAC alone group (*p* < 0.05). **Srivastava S. et al. (2017)** did not observe a statistically significant difference in all the parameters in VSS at baseline in both the groups but a gradual reduction in height, vascularity, pliability, and pigmentation at every successive assessment in all the groups, which was maintained till the final evaluation.^8^

POSAS scores (observer and patient scale) were significantly reduced from the baseline in both groups in our study, but were significantly lower in the combination group at 12‐week follow‐up (*p* < 0.05). Till date, no study has used the POSAS score for comparing the efficacy of combination therapy with TAC and 5‐FU versus TAC alone. **KE Hietanen et al. (2018)** used the POSAS score to compare the efficacy of TAC and 5‐FU injections for the treatment of keloids and found the difference in the POSAS evaluation (observer and patient) between baseline and 6 months as statistically significant (*p* < 0.05).^20^ A recent prospective study by **Reinholz M et al. (2020)** used the POSAS score to see the treatment response of 5‐FU in combination with crystalline TAC suspension in keloids.^21^

In this study, 27 (87%) patients in the TAC group developed adverse effects compared to 14 (44%) in the combination group which is similar to the study by **F.A. Khalid et al. (2018)**, who found significantly more overall adverse effects in the TAC alone group (35.2%) than in the combination group (14%).^19^ Among the adverse events, a statistically significant difference was observed in telangiectasis (reported in 51.6% of TAC patients vs. 3.1% combination group). The higher rate of telangiectasis compared to that reported by **F.A. Khalid et al. (2018)**, (23.5% in the TAC alone and 3.5% in the combination group),^19^ ^.^ can be attributed to the higher concentration of TAC in our study. Telangiectasia (reported in 51.6% of TAC patients vs. 3.1% combination group) was negated by the effect of 5‐FU and this maybe one of the reasons for less telangiectasia (and adverse effects overall) in the combination group. Interestingly, our study showed hyperpigmentation in contrast to the hypopigmentation in the TAC alone group in the F.A. Khalid et al. (2018) study, whereas hyperpigmentation was also less observed in the combination group.^19^ No cases developed ulceration in our study whereas **F.A. Khalid et al. (2018)** found ulcerations in 8.8% of cases in the combination group, which can be explained by the lower concentration of 5‐fluorouracil (25 mg/mL) used in our study.^19^

Similarly, no cases developed lesion atrophy in both of our study groups which is in contrast to the study by **F.A. Khalid et al. (2018)** where atrophy was seen in the TAC alone group in 17.6% of cases.^19^ This might be due to the uniform drug distribution in the scar by definite injection and dose protocol and longer interval in our study (2 weekly vs. 1 weekly).

In our study, no systemic effects were seen which supported the previous studies.^14^, ^18^, ^19^ The adverse effects were minimal and transient and did not require any further treatment.

Being a single‐centred study with no long‐term follow‐up is our major limitation as it hinders predicting recurrence. Hence, we would recommend multicentred, large‐sample sized, double‐blinded, and longer period studies.

## 5 CONCLUSION

Our study found that intralesional triamcinolone acetonide in combination with 5‐fluorouracil was more efficacious in terms of reduction in scar height, scar scores, and subjective improvement as reported by patients and observers and provide more rapid response with fewer side effects.

## CONFLICT OF INTEREST STATEMENT

The authors declare no conflicts of interest.

## AUTHOR CONTRIBUTIONS

**Ripala Acharya**: Conceptualization (lead); data curation (lead); formal analysis (lead); funding acquisition (equal); investigation (lead); methodology (equal); project administration (lead); resources (lead); software (lead); validation (lead); visualization (lead); writing – original draft (lead); writing – review & editing (lead). **Sudha Agrawal**: Conceptualization (lead); formal analysis (equal); funding acquisition (equal); investigation (equal); methodology (lead); supervision (lead); validation (equal); visualization (equal). **Dhan Keshar Khadka**: Conceptualization (supporting); methodology (supporting); project administration (supporting); supervision (supporting); visualization (supporting). **Aashish Raj Pant**: Data curation (equal); formal analysis (equal); writing – review & editing (lead).

## ETHICS STATEMENT

Ethical approval from the Institutional Review Committee, BPKIHS (Code no: IRC/1890/020) and Nepal Health Research Council (ERB Protocol No:721/2020MT). The trial was registered in ClinicalTrial.gov (NCT04812626).

## PATIENT CONSENT

Written patient consent was obtained for publication.

## Supporting information

Supporting Information S1

## Data Availability

All data are incorporated in the article and its online supplementary material.
