# Supplementary material for: Efficacy and safety of intralesional triamcinolone acetonide alone and its combination with 5‐ fluorouracil in keloids and hypertrophic scars: Randomized, parallel group, and double blinded trial
Source: Skin Health Dis. 2024 Aug 24;4(5):e450. doi: 10.1002/ski2.450 (PMC11442070; doi:10.1002/ski2.450)
Supplement: Supplementary file 1 — Supporting Information S1 [file SKI2-4-e450-s001.docx]

Efficacy and safety of intralesional triamcinolone acetonide alone and its combination with 5- fluorouracil in keloids and hypertrophic scars: Randomized, Parallel Group, Double-Blinded Trial

# Acharya R1, Agrawal S2, Khadka DK^3^, Pant AR^4^

1,2,3Department of Dermatology and Venereology, B.P Koirala Institute of Health Sciences, Dharan, Nepal

^4^Department of Oculofacial Plastic Surgery, Mechi Eye Hospital, Jhapa, Nepal

**Corresponding author**

Ripala Acharya, Department of Dermatology and Venereology, B.P Koirala Institute of Health Sciences, Dharan, Nepal.

Email: [ripalaacharya@gmail.com](mailto:ripalaacharya@gmail.com)


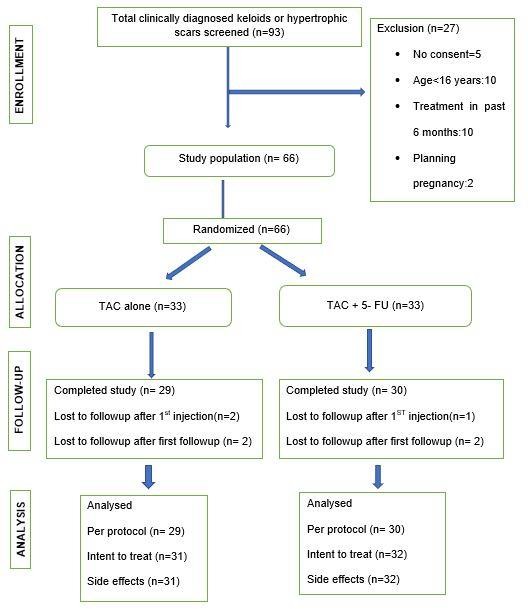


# Figure S1: CONSORT diagram

(i)

(ii)

**Figure S2: Mean height in each treatment groups-(i)Intent to treat, (ii) Per-protocol**


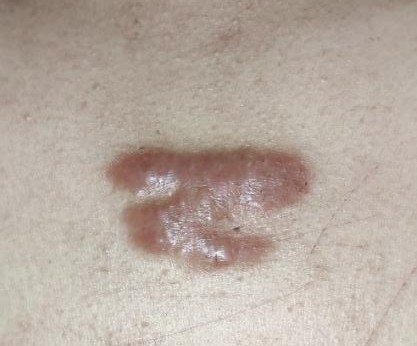

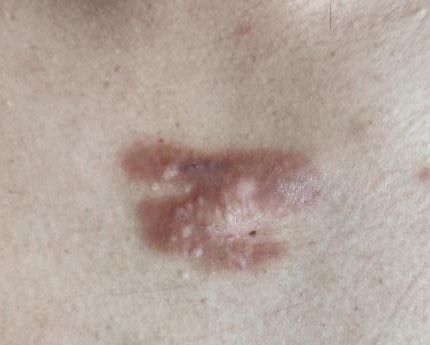

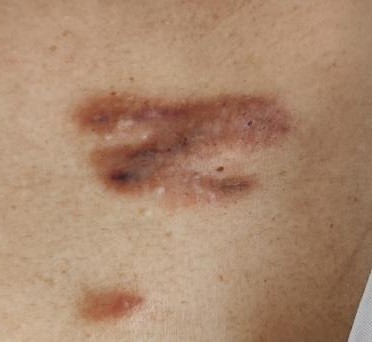
i ii


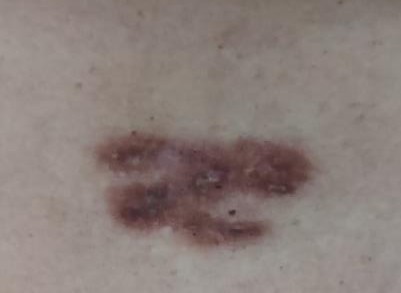

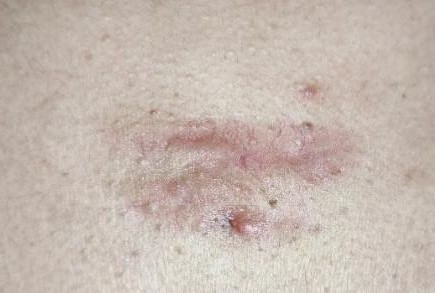

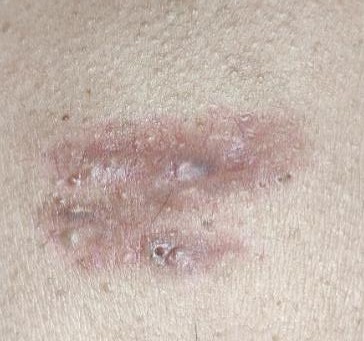
 iv iii

vi

v


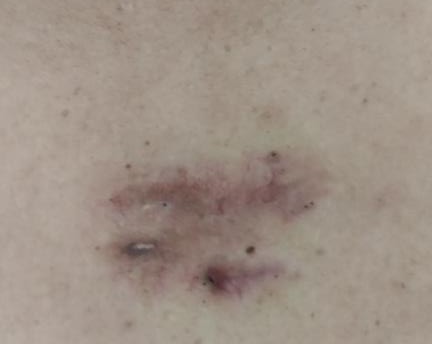


**Figure S3**:Scar treated with TAC+5-FU: i; week(wk) 0, ii:wk 2, iii:wk 4, iv:wk 6, v:wk 8, vi:wk10, vii:wk 12; Shows:

Height reduction:90%;VSS: 9🡪6;POSAS observer:33🡪16, POSAS patient:27🡪8;Patient reported improvement:99%;Observer reported improvement:90%

vii


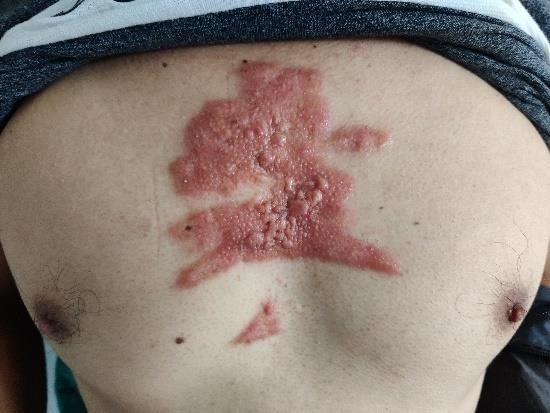

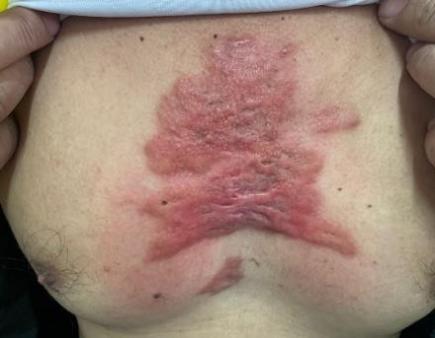


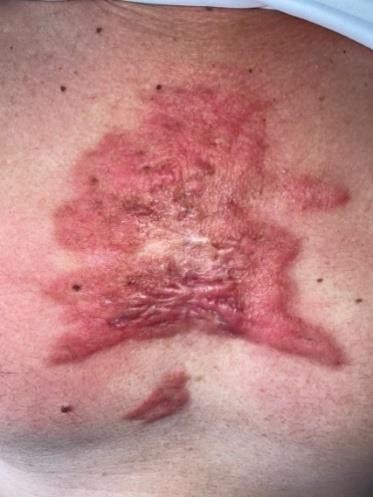
i


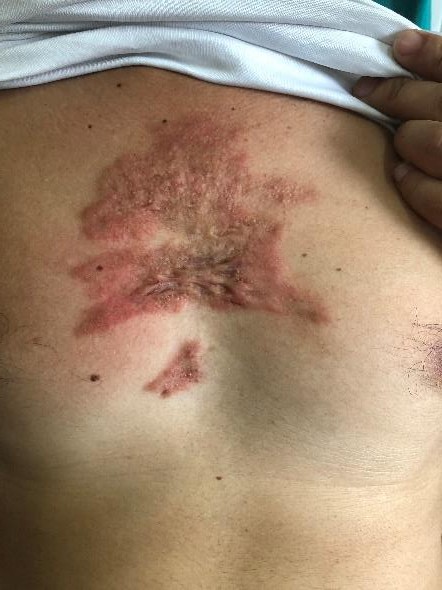


ii

iii iv


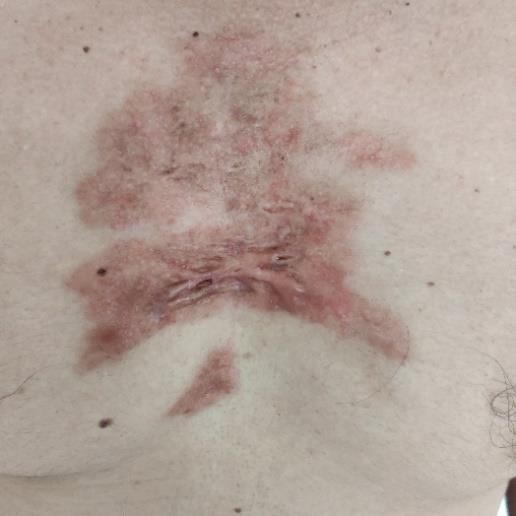

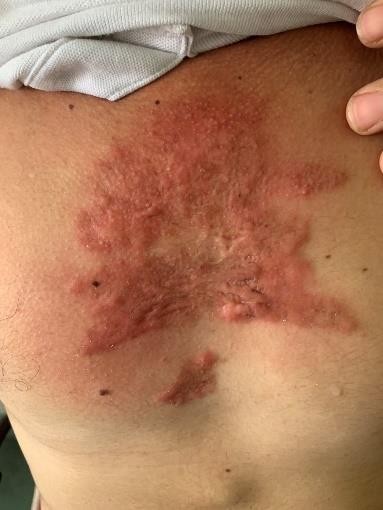
v vi


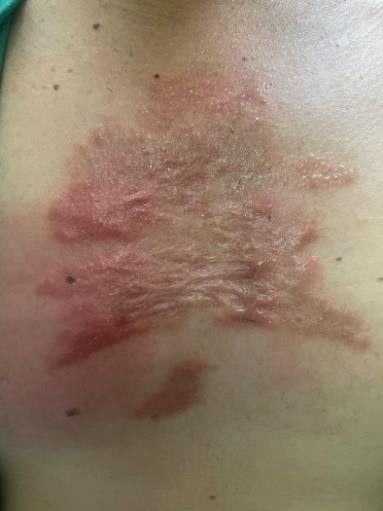


vii

**Figure S4**: Scar treated with TAC+5-FU: i; week(wk) 0, ii:wk 2, iii:wk 4, iv:wk 6, v:wk 8, vi:wk10, vii:wk 12; Shows:

Height reduction:80%;VSS: 9🡪7;POSAS observer:47🡪17, POSAS patient:60🡪13;Patient reported improvement:90%;Observer reported improvement:60%


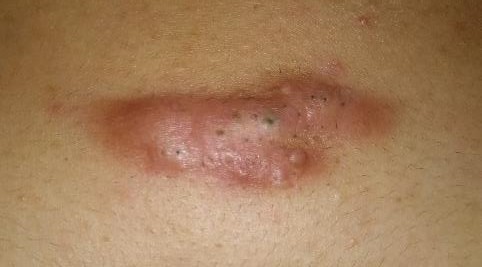


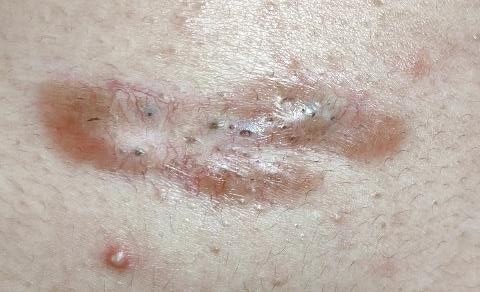

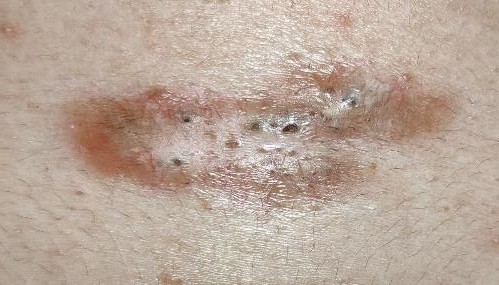

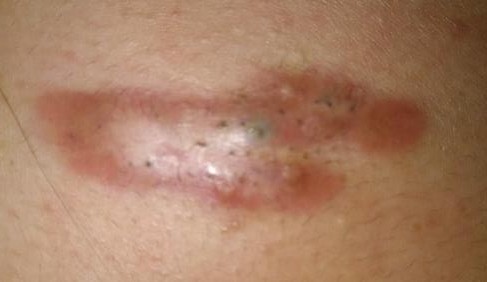
i ii

iii iv


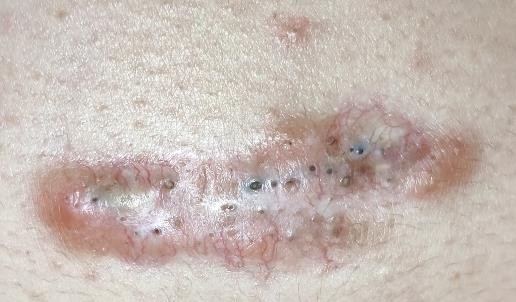


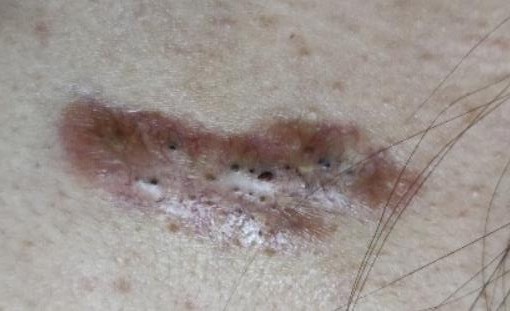
vi

v


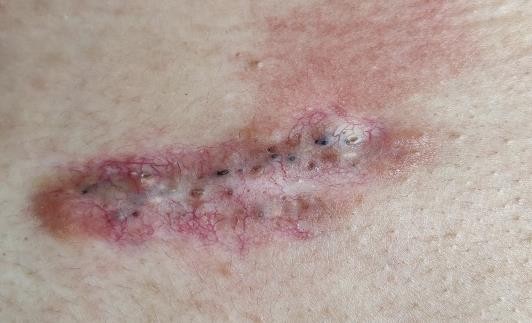


**Figure S5**: Scar treated with TACalone i:wk 0, ii:wk 2, iii:wk 4, iv:wk 6, v:wk 8, vi:wk10,

vii:wk 12

Height reduction:60%;VSS:9🡪7;POSAS observer: 37🡪22,

patient:30🡪22;Patient improvement:50%;Observer improvement:55%

POSAS

reported reported

vii


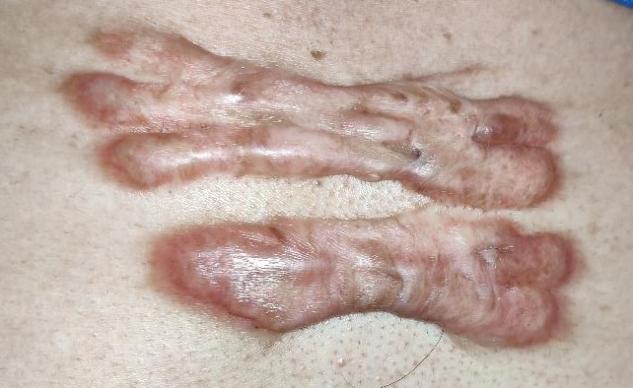

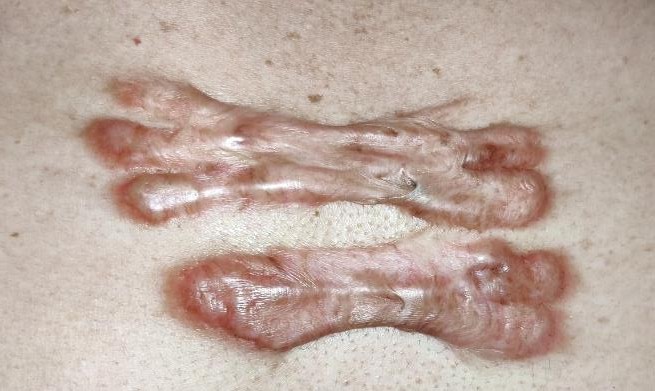


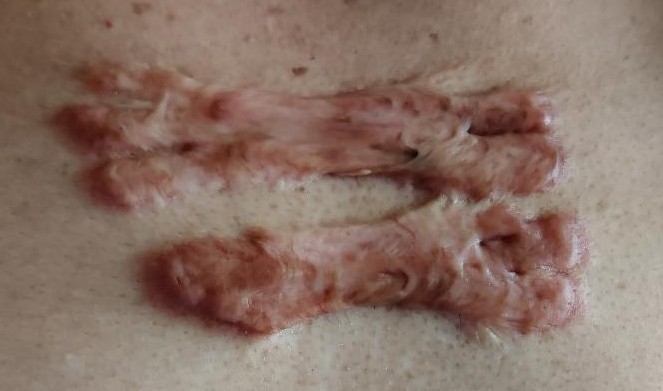
i ii


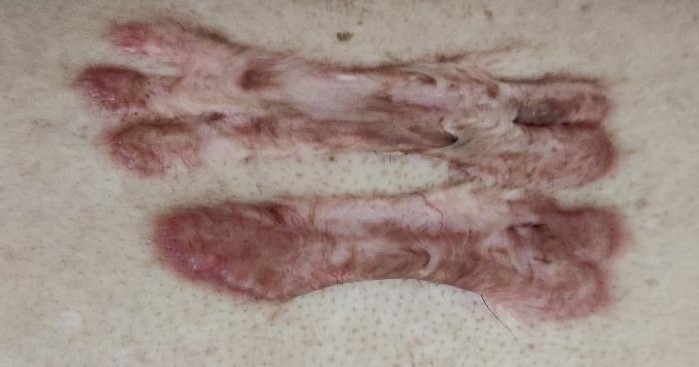


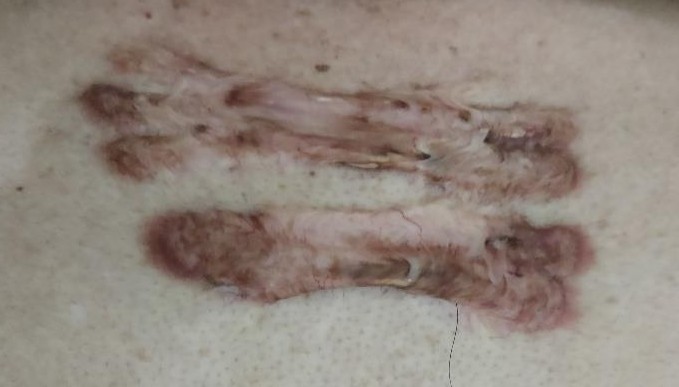

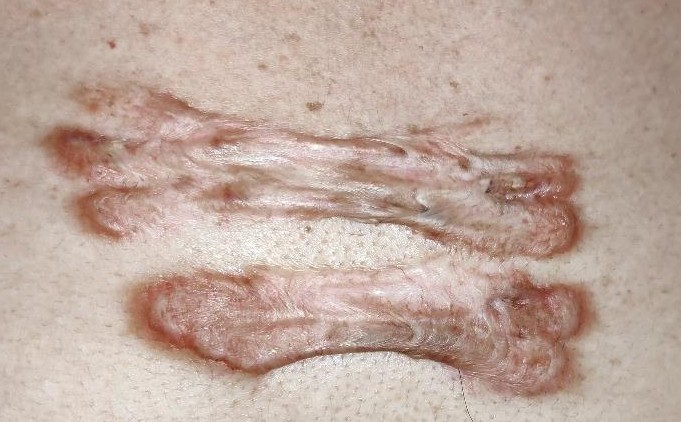
iii iv

v vi


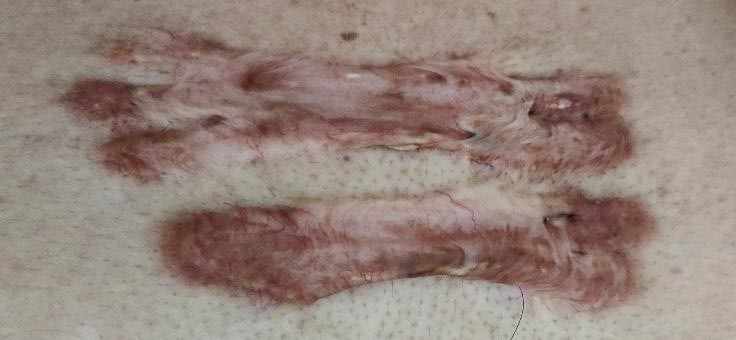


**Figure S6**: Scar treated with TACalone; i:wk 0, ii:wk 2, iii:wk 4,

iv:wk 6, v:wk 8, vi:wk10, vii:wk 12

Height reduction:40%; VSS: 10🡪7; POSAS observer:44🡪20, POSAS patient:46🡪23; Patient reported improvement:50%; Observer reported improvement:50%

vii

# Table S1: Baseline characteristics of keloids and hypertrophic scars patients

| **Characteristics** | **Group** | | **p-value** |
| --- | --- | --- | --- |
|  | **TAC alone** | **TAC+5-FU** |  |
| **Age(years)**, Mean±SD  Median(Min-Max) | 29.10±10.959  28(16-53) | 25.88±8.308  23(16-47) | 0.335** |
| **Sex, n (%)**  Female  Male | 14(45)  17(55) | 9(28)  23(72) | 0.160* |
| **Duration(months)** Mean±SD  Median(Min-Max) | 28.81±39.058  12(3-180) | 40.02±42.712  24(4-144) | 0.413** |
| **Cause/Precipitating factors,n(%)**  Acne  Trauma  Unknown  Piercing  Surgery  Varicella  Burn | 8(25.8)  7(22.6)  5(16.1)  4(12.9)  4(12.9)  2(6.5)  1(3.2) | 19(59.4)  5(15.6)  2(6.3)  3(9.4)  0(0)  0(0)  3(9.4) | NA |
| **Pruritus,n(%)**  No Yes | 4(13)  27(87) | 5(15.6)  27(84.4) | 1.00*** |
| **Pain, n(%)**  No Yes | 23(74.2)  8(25.8) | 22(69)  10(31) | 0.633* |
| **Past treatment, n(%)**  No Yes | 25(80.6)  6(19.4) | 22(69)  10(31) | 0.278* |
| **Past treatment type group, n(%)**  Intralesional steroids  Others | 4(67)  2(33) | 8(80)  2(20) | 0.604*** |
| **Type of scars, n(%)**  Keloid  Hypertrophic scar | 28(90.3)  3(9.7) | 29(90.6)  3(9.4) | 1.000*** |
| **Number of site**s  Mean±SD  Median(Min-Max) | 1.03±0.180  1(1-2) | 1.09±0.296  1(1-2) | 0.321** |
| **Number of lesions**  Mean  Median(Min-Max) | 2.55±3.335  1(1-15) | 3.25±2.962  2.5(1-12) | 0.055** |
| **Initial height(mm)**  Mean±SD  Median(Min-Max) | 7.0 ±3.06  6(3-17) | 5.41±1.68  5(3-10) | **0.018**** |
| **Initial VSS**  Mean±SD  Median(Min-Max) | 9.06±1.65  9(3-11) | 9.22±0.90  9(6-11) | 0.655** |
| **Initial POSAS(observer)**  Mean±SD  Median(Min-Max) | 33.55±8.74  35(12-51) | 34.59±5.40  33(27-47) | 0.923** |
| **Initial POSAS(patient)**  Mean±SD  Median(Min-Max) | 33.74 ±8.43  34(15-50) | 37.69±8.68  37.5(25-60) | 0.144** |

* Pearson chi-square test , ** Mann-Whitney U test, ***Fischer’s

**Table S2: Comparison of each side effects between the treatment groups**

| **Side effects** | | **Group of Patient** | | | | **P-value*** |
| --- | --- | --- | --- | --- | --- | --- |
|  |  | **TAC alone(N=31)** | | **TAC+5-FU**  **(N=32)** | |  |
|  |  | **Frequency** | **%** | **Frequency** | **%** |  |
| Hyperpigmentation | Yes | 14 | 45.2 | 13 | 40.6 | 0.716 |
|  | No | 17 | 54.8 | 19 | 59.4 |  |
| Telangiectasia | Yes | 16 | 51.6 | 1 | 3.1 | <0.001 |
|  | No | 15 | 48.4 | 31 | 96.9 |  |
| Pain | Yes | 0 | 0 | 1 | 3.1 | NA |
|  | No | 31 | 100 | 31 | 96.1 |  |

*Pearson chi square test
